# Randomized controlled clinical trial of Shenzhuo Formula in the treatment of macroalbuminuria in diabetic kidney disease and its inflammation-modulating mechanisms

**DOI:** 10.1093/pcmedi/pbaf031

**Published:** 2025-11-14

**Authors:** Anzhu Wang, Yishan Huang, Yu Wei, Lili Zhang, Hongdong Chen, Xiaoqing Wang, Zhimei Cui, Bin Wang, Wei Liu, Chao Chen, Ye Lei, Zhonghua Zheng, Yan Wei, Jia Mi, Keda Lu, Ying Zhang, Xiaolin Tong, Linhua Zhao

**Affiliations:** Key Laboratory of Endocrine Glucose & Lipids Metabolism and Brain Aging, Ministry of Education; Department of Endocrinology, Shandong Provincial Hospital Affiliated to Shandong First Medical University, Jinan 250021, China; Metabolic Disease Institute, Guang’anmen Hospital, China Academy of Chinese Medical Sciences, Beijing 100053, China; Metabolic Disease Institute, Guang’anmen Hospital, China Academy of Chinese Medical Sciences, Beijing 100053, China; Metabolic Disease Institute, Guang’anmen Hospital, China Academy of Chinese Medical Sciences, Beijing 100053, China; Department of Endocrinology, Beijng Hepingli Hospital, Beijing 100013, China; Baoding Traditional Chinese Medicine Hospital, Baoding 071000, China; Shijiazhuang Traditional Chinese Medicine Hospital, Shijiazhuang 050051, China; Zouping Country Hospital of Traditional Chinese Medicine, Zouping 256200, China; The Central Hospital of Yongzhou, Yongzhou 425006, China; Shantou Traditional Chinese Medicine Hospital, Shantou 515031, China; The Second Affiliated Hospital of Shaanxi University of Traditional Chinese medicine, Xianyang 712000, China; Zhengzhou Traditional Chinese Medicine Hospital, Zhengzhou 450007, China; Hangzhou Hospital of Traditional Chinese Medicine, Hangzhou 310007, China; Affiliated Hospital of Changchun University of Chinese Medicine, Changchun 130021, China; Zhejiang Provincial Hospital of Chinese Medicine, Hangzhou 310005, China; Beijing University of Chinese Medicine, Beijing 100029, China; Metabolic Disease Institute, Guang’anmen Hospital, China Academy of Chinese Medical Sciences, Beijing 100053, China; Metabolic Disease Institute, Guang’anmen Hospital, China Academy of Chinese Medical Sciences, Beijing 100053, China

**Keywords:** diabetic kidney disease, traditional Chinese medicine, Shenzhuo Formula, macroalbuminuria

## Abstract

**Background:**

Shenzhuo Formula (SZF), a modified Didang Tang, is used for diabetic kidney disease (DKD), though high-quality evidence is limited.

**Methods:**

In a randomized, double-blind, double-dummy, active-controlled, multicenter trial, irbesartan (IRB) was the control. A Bayesian model assessed efficacy. Mechanistic studies included Olink inflammation proteomics, single-cell RNA sequencing (scRNA-seq) of KK-Ay mouse kidneys, and *in vivo* experiments.

**Results:**

A total of 120 DKD patients with macroalbuminuria were randomized (SZF *n* = 57, IRB *n* = 63). At 24 weeks, 24 h urinary total protein change was −0.03 (−0.24 to 0.18) g/24 h in the SZF group and 0.08 (−0.30 to 0.14) g/24 h in the IRB group (*P* = 0.61). Estimated glomerular filtration rate improved with SZF by 5.91 (1.80 to 10.01) mL/min/1.73m² but declined with IRB by −1.67 (−5.18 to 1.84) mL/min/1.73m² (*P* < 0.01). Serum creatinine decreased with SZF by −5.15 (−9.73 to −0.56) μmol/L but increased with IRB by 3.39 (−0.84 to 7.61) μmol/L (*P* < 0.01). Traditional Chinese medicine syndrome response was higher with SZF (89.47% vs. 63.49%, *P* < 0.01). Safety and metabolic parameters were comparable. Bayesian analysis favored SZF for renal benefit. Mechanistically, SZF downregulated CX3CL1 in endothelial cells and MCP-1 in mesangial and tubular cells, suggesting anti-inflammatory effects restoring endothelial function and attenuating fibrosis.

**Conclusions:**

SZF matched IRB in proteinuria reduction but was superior in preserving renal function and improving traditional Chinese medicine symptoms in DKD, with good safety. Benefits may involve suppression of CX3CL1/MCP-1-mediated inflammation.

## Introduction

Diabetic kidney disease (DKD) is a chronic kidney disease (CKD) caused by diabetes mellitus (DM), and its rising prevalence parallels the rapid global increase in DM cases [1, 2]. Clinically, DKD is characterized by persistent increases in albuminuria and/or a progressive decline in estimated glomerular filtration rate (eGFR), eventually leading to end-stage renal disease (ESRD). Globally, ∼30% to 50% of ESRD cases are attributed to DKD [3, 4]. DKD has become the leading cause of ESRD, imposing significant economic and medical burdens on healthcare systems worldwide [5].

Albuminuria is a key marker for the clinical diagnosis, staging, and risk stratification of DKD, as well as an important biomarker for assessing disease progression. Once macroalbuminuria develops, the progression of DKD to ESRD occurs at a much faster rate than other types of CKD [6]. The primary effective drugs for reducing albuminuria in DKD are angiotensin-converting enzyme inhibitor (ACEI) and angiotensin receptor blocker (ARB), which can effectively lower albuminuria and delay renal function decline. However, most patients still face high residual risk [7, 8]. Moreover, the side effects of ACEI/ARB—such as hypotension, angioedema, dry cough, transient increases in serum creatinine (SCr), and hyperkalemia—often compromise long-term adherence. There is particular debate regarding the use of ACEI/ARB drugs in patients with SCr levels >265 μmol/L [9].Thus, an effective and safe risk–benefit therapy is needed for DKD patients.

DKD falls under the category of “Xiaoke nephropathy” in traditional Chinese medicine (TCM), with its core pathogenesis being “chronic illness entering the meridians, causing kidney meridian stagnation and obstruction.” [10]. Ancient texts from the Han Dynasty (206 BCE to 220 CE) document that TCM could treat albuminuria and edema, which are the main clinical manifestations of DKD [11]. Over the long course of medical practice, TCM has developed herbal treatments for DKD, with Da Huang (*Rheum palmatum L*.) and Shui Zhi (*Whitmania pigra Whitman*) being representative medicines. The classic formula “Didang Tang” was first recorded in the Eastern Han Dynasty in the medical text Shang Han Lun and has been widely used for >1 800 years. In recent decades, multiple clinical studies have employed Didang Tang to treat DKD [12–16]. Shenzhuo Formula (SZF) is an improved version of the classic formula Didang Tang. It works by tonifying qi, activating blood circulation, and unblocking the meridians to treat DKD patients. Previous real-world clinical studies have demonstrated that SZF effectively reduces 24 h urinary total protein (24hUTP) in DKD patients and delays disease progression [17, 18]. However, there are no randomized clinical trials directly comparing the efficacy and safety of TCM versus ACEI/ARB in reducing albuminuria risk among DKD patients. Building upon the initial clinical findings and experimental research, a prospective, multicenter, double-blind, double-dummy, active-controlled trial is planned. This trial aims to evaluate the benefits and safety of SZF compared to irbesartan (IRB) in treating DKD.

Traditionally, the pathogenesis of DKD has been attributed to the combined effects of metabolic and hemodynamic factors. In recent years, accumulating evidence has suggested that DKD is also a chronic inflammatory disease. Persistent inflammatory responses contribute to progressive fibrosis, structural damage, and functional loss, thereby accelerating the proliferation and fibrotic transformation of glomerular and tubulointerstitial cells [19]. Based on this concept, we employed Olink inflammation proteomics and single-cell RNA sequencing (scRNA-seq) to identify the potential anti-inflammatory mechanisms of SZF.

## Materials and methods

### Study design

This study was a prospective, multicenter, double-blind, double-dummy, active-controlled randomized clinical trial (No. ChiCTR-ICR-15006311). A total of 11 clinical centers were involved, including Guang’anmen Hospital of China Academy of Chinese Medical Sciences, Baoding Traditional Chinese Medicine Hospital, Shijiazhuang Traditional Chinese Medicine Hospital, Zouping County Traditional Chinese Medicine Hospital (Shandong Province), Yongzhou Central Hospital (Hunan Province), Shantou Traditional Chinese Medicine Hospital, The Second Affiliated Hospital of Shaanxi University of Traditional Chinese Medicine, Zhengzhou Traditional Chinese Medicine Hospital, Hangzhou Traditional Chinese Medicine Hospital, Zhejiang Provincial Traditional Chinese Medicine Hospital, and the Affiliated Hospital of Changchun University of Traditional Chinese Medicine. The protocol was approved by the Ethics Committee of Guang’anmen Hospital (No. 2015EC038). The trial design and statistical analysis plan were informed by previously published studies [20, 21].

The study was conducted in accordance with the principles of the Declaration of Helsinki [22] and adhered to the guidelines of Good Clinical Practice to ensure the proper application of TCM in clinical settings. Moreover, the trial protocol was designed following the SPIRIT 2013 statement for clinical trial protocols [23]. Written informed consent was obtained from all participants before enrollment. This study followed Consolidated Standards of Reporting Trials (CONSORT) reporting guidelines.

### Study population

The diagnostic criteria were based on the diagnostic standards for T2DM as defined by the 1999 World Health Organization (WHO) [24] and the 2013 American Diabetes Association (ADA) guidelines [25]. The diagnostic criteria for DKD followed the Kidney Disease Outcome Quality Initiative (K/DOQI) guidelines published by the National Kidney Foundation in 2007 and 2012 [26]. The deficiency of qi with blood stasis syndrome in TCM adhered to the standards outlined in the Guidelines for Clinical Research on New Chinese Medicines [27] and the Evidence-Based Clinical Practice Guidelines for Traditional Chinese Medicine in Diabetes [28].

Inclusion criteria: (i) participants meeting the diagnostic criteria for DKD; (ii) participants meeting the TCM diagnostic criteria for qi deficiency and blood stasis syndrome; (iii) aged between 18 and 80 years; (iv) informed consent of participants; (v) hemoglobinA1c (HbA1c) ≤ 8.0%; (vi) 0.5 g < 24hUTP ≤ 3 g; (vii) SCr ≤ 133 μmol/l (1.5 mg/dL); (viii) controlled hypertension [blood pressure (BP) ≤ 140/90 mmHg].

Exclusion criteria: (i) type 1 diabetes mellitus; (ii) poorly controlled blood glucose (HbA1c > 8%); (iii) use of potassium-sparing diuretics; (iv) severe anemia (hemoglobin < 60 g/L); (v) serum albumin < 35 g/L; (vi) history of severe cardiovascular or cerebrovascular disease; (vii) kidney damage caused by other factors, such as drug-induced kidney injury, IgA nephropathy, or hyperuricemic nephropathy; (viii) pregnant or breastfeeding women, or women planning to conceive or without contraception plans; (ix) allergy to Chinese herbal components or known allergic constitution; (x) participants with psychiatric disorders; (xi) participation in another clinical trial within 1 month prior to enrollment or ongoing participation in another clinical trial; (xii) any other conditions or comorbidities deemed by the participants to reduce eligibility or complicate trial participation, such as unstable living conditions or frequent job relocations leading to a high risk of loss to follow-up; (xiii) impaired liver function [alanine aminotransferase (ALT) or aspartate aminotransferase (AST) levels exceeding 2.5 times the upper limit of normal].

### Sample size calculation

Sample size has been calculated based on the primary endpoint and average change of 24hUTP from baseline to week 24. Previous studies suggest a mean difference of 0.2 g between groups, and the same standard deviation (SD) of 0.3 g for each group. We expect that a total of 100 subjects will be needed to uncover any difference between groups with at least a power of 90%, controlling the type I error rate at 0.05. Considering a dropout rate of 20%, the target sample size will be 120 [21].

### Randomization and blinding

Eligible patients were randomly assigned to groups at a 1:1 ratio using a central permuted block randomization method (block size of 4). The SZF group received SZF along with a placebo for IRB, while the IRB group received a placebo for SZF along with IRB. Randomization numbers were independently generated by a statistician at the Institute of Clinical Basic Medicine, China Academy of Chinese Medical Sciences, using SAS 9.4.

A comprehensive blinding procedure was implemented to ensure that treatment allocations remained confidential before randomization, ensuring that both investigators and participants were blinded to the treatment assignments. To maintain the integrity of blinding, the SZF and its placebo were identical in shape, odor, and color, and both had a consistent appearance when dissolved in water. Similarly, the IRB capsules and their placebo were indistinguishable in smell and appearance after opening the capsules, making it nearly impossible for participants to differentiate between them. All the participants, researchers, and statisticians were blinded to the study protocols.

### Interventions

This study included a 2-week run-in period and a 24-week intervention period. During the entire study, both groups received standard baseline treatments. According to the guidelines from the ADA, all participants were treated to maintain BP ≤ 140/90 mmHg [25]. Recommended antihypertensive drugs included non-dihydropyridine calcium channel blockers or beta-blockers. Standard care also encompassed glycemic control: fasting blood glucose (FBG) levels were maintained at ≤ 7.8 mmol/L, and 2 h postprandial glucose levels at ≤ 11.1 mmol/L throughout the trial. Participants were advised not to alter their routine medications for chronic diseases unless deemed necessary.

Eligible participants were randomized into either the SZF group or the IRB group. SZF is composed of six natural Chinese medicines (supplementary Table 1, see online supplementary material). Participants in the SZF group were administered SZF granules (packaging: 56 sachets/box, manufacturer: Jiangyin Tianjiang Pharmaceutical Co., Ltd., Wuxi, China), two sachets daily taken orally, one in the morning and one in the evening. They also received IRB placebo capsules (packaging: 75 mg × 56 capsules/bottle, manufacturer: Bailing Pharmaceutical Co., Ltd., Guizhou, China and Jiulong Pharmaceutical Co., Ltd. Beijing, China), with a daily dose of 150 mg taken orally in the morning. Participants in the IRB group were administered SZF placebo granules (packaging: 56 sachets/box, manufacturer: Jiangyin Tianjiang Pharmaceutical Co., Ltd., Wuxi, China) and IRB capsules (packaging: 75 mg × 56 capsules/bottle, manufacturer: Hongsheng Pharmaceutical Co., Ltd., Hangzhou, China). The production processes of all study drugs were subject to quality control to ensure consistency in purity, microbial content, weight, and other physical properties. Certificates of quality were issued by the manufacturers for the investigational products used in both the SZF and IRB groups.

Investigational medicinal products (IMPs) were distributed only to participants who had provided written informed consent and successfully met all screening inclusion and exclusion criteria. At each site, only qualified personnel were authorized to dispense the medications to participants. They ensured that the IMPs were securely sealed and stored in a cool, dry, and locked location. A composition analysis of the SZF is provided in supplementary Fig. 1, see online supplementary material.

During the trial, the use of medications that could influence albuminuria or kidney function was prohibited. These included TCM drugs aimed at invigorating qi or promoting blood circulation, potassium-sparing diuretics, and other ACEI/ARB. Follow-up visits were conducted every 4 weeks, during which participants underwent assessments according to the study protocol. At each visit, all returned and unused/empty IMP packages were accounted for, and details such as counts and return dates were recorded in the IMP log. Participants were also advised not to alter their routine medications for chronic diseases unless necessary. All investigators were experienced in managing DKD patients and underwent comprehensive training on the study protocol prior to its initiation. Investigators were instructed to document any additional or alternative medications or therapies in the case report form, including details such as drug names, dosages, and duration of administration.

### Outcome measures

The primary endpoint was the change in 24hUTP from baseline to week 24. Secondary endpoints included: (i) changes in SCr and eGFR from baseline to week 24; (ii) changes in FBG from baseline to week 24; (iii) changes in blood lipids from baseline to weeks 12 and 24, including total cholesterol (TC), triglycerides (TG), high-density lipoprotein cholesterol (HDL-C), and low-density lipoprotein cholesterol (LDL-C); (iv) changes in HbA1c from baseline to weeks 12 and 24; (v) changes in BP from baseline to week 24; (vi) improvements in TCM symptoms from baseline to week 24, assessed using a symptom scoring method based on the nimodipine approach. Efficacy index = [(score before treatment − score after treatment)/score before treatment] × 100%. The evaluation criteria are as follows. (i) Clinical control: most symptoms disappear after treatment (efficacy rate ≥ 95%). (ii) Significant effect: most clinical symptoms show marked improvement (efficacy rate ≥ 70%). (iii) Effective: symptoms are alleviated (efficacy rate ≥ 30%). (iv) Ineffective: symptoms show no significant improvement or worsen (efficacy rate < 30%).

Safety assessments will be conducted at baseline, week 12, and week 24, including the following: (i) routine blood tests, urinalysis, and stool tests; (ii) electrocardiogram; (iii) liver function tests, including ALT and AST; (iv) renal function tests, including blood urea nitrogen and β2-microglobulin; (v) adverse events (AEs): recorded at each visit, including signs, symptoms, and other illnesses. Each AE will be categorized as mild, moderate, or severe, and its relationship to the investigational drug will be assessed. Serious adverse events will be reported to the principal investigator and the ethics committee within 24 h. All AEs will be recorded, monitored, and treated until resolution. Additional necessary diagnostic tests, such as computed tomography and ultrasound will be performed if symptoms meet the criteria for an AE. To ensure compliance with the study protocol, one to six unscheduled visits will be conducted during the study period across all participating centers.

### Olink inflammation-targeted proteomics

The Olink Target 96 Inflammation Panel was used to detect inflammation-related proteins in plasma samples from 28 participants (baseline group: *n* = 14; post-intervention group: *n* = 14). A total of 92 inflammatory proteins were measured. The Olink assay is based on a proximity extension analysis, in which paired antibodies bind the target protein and are conjugated to complementary DNA probes. When the probes are in close proximity, they hybridize and are extended to form a qPCR(Quantitative polymerase chain reaction) template, followed by qPCR amplification and quantification. Protein expression was presented as log₂-transformed normalized protein expression values, batch-effect corrected. Differentially expressed proteins were defined by |log₂FC(Fold change)| > 0.2, paired *t*-test, and Benjamini–Hochberg adjustment (*P* < 0.05). Volcano plots and heatmaps were generated using R.

### Animal source and ethical statement

All animal experiments were approved by the Animal Ethics Committee of Guang’anmen Hospital, China Academy of Chinese Medical Sciences (Approval No. IACUC-GAMH-2025–002), and were conducted in strict accordance with the “Administrative Measures for Laboratory Animal Ethics Review” and the NIH Guide for the Care and Use of Laboratory Animals. Male C57BL/6J mice (6–7 weeks) and KK-Ay diabetic mice were purchased from Beijing Huafukang Bioscience Co., Ltd. (license No. SCXK [Jing] 2024–0003).

### Housing and diet

All animals were housed individually in a specific pathogen-free barrier facility, with a 7-day acclimation period. Environmental conditions were maintained at 22–25°C, relative humidity 55% ± 5%, and a 12 h/12 h light–dark cycle (lights on 07:00–19:00). KK-Ay mice were fed a high-fat diet (w/w: 17.5% protein, 17.9% fat, 48.5% carbohydrate), while C57BL/6J mice received a standard chow diet. Food and water were provided *ad libitum*.

### Experimental groups and treatment protocol

Four groups of mice (*n* = 6/group) were established: (i) control: C57BL/6J mice receiving vehicle (sterile water, gavage) for 12 weeks; (ii) DKD model (KK-Ay): KK-Ay mice receiving vehicle for 12 weeks; (iii) positive control (KK-Ay + IRB): KK-Ay mice gavaged with IRB (22.75 mg·kg⁻¹·day⁻¹) for 12 weeks; (iv) SZF treatment (KK-Ay + SZF): KK-Ay mice fed high-fat diet plus SZF extract (18 g crude drug·kg⁻¹·day⁻¹, gavage) for 12 weeks. All interventions were administered at a fixed daily time, with body weight measured weekly to adjust gavage volume (10 mL·kg⁻¹).

### Drug preparation and administration

IRB was dissolved in sterile water at 0.1 mg·mL⁻¹ and administered at 22.75 mg·kg⁻¹·day⁻¹. SZF extract was prepared by the Pharmacy Department of Guang’anmen Hospital using traditional decoction, stored at 4°C protected from light, and reconstituted in sterile water to 1.8 g crude drug·mL⁻¹ prior to administration (18 g crude drug·kg⁻¹·day⁻¹). After 16 weeks of treatment, mice were fasted for 12 h, anesthetized with 1% sodium pentobarbital (50 mg·kg⁻¹, intraperitoneal injection), and blood and kidney tissues were collected.

### Serum and urine biochemistry

Serum and 24 h urine were analyzed using a Toshiba TBA-120FR automated biochemical analyzer (Toshiba Medical Systems, Japan) with reagents from Beijing Beijian New Source Biotechnology Co., Ltd.

### Single-nucleus RNA sequencing of mouse kidneys

Fresh kidney tissues were minced (< 2 mm) and homogenized in ice-cold Luria–Bertani buffer (CapitalBio) containing 1% bovine serum albumin, 1 mM dithiothreitol, and RNase inhibitor. Following lysis, filtration, and density-gradient centrifugation, nuclei were isolated, washed, and counted (viability > 80%). Single-nucleus suspensions were loaded into a 10x Genomics Chromium platform (Single Cell 3′ v3.1 chemistry) targeting 10 000 nuclei. Reverse transcription and cDNA amplification were followed by library preparation and Illumina NovaSeq 6000 sequencing (PE150, ∼50 000 read pairs/nucleus). Sequencing data were processed using Cell Ranger 6.0 with the GRCm39 reference genome to generate the gene expression matrix. Nuclei with <200 detected genes or with mitochondrial gene content >25% were filtered out. CellBender was applied to remove ambient RNA, and DoubletFinder was used to eliminate doublets. Samples were integrated using the anchor-based integration method in Seurat 4.0, followed by normalization, principal component analysis, clustering, and uniform manifold approximation and projection (UMAP) visualization. Differentially expressed genes were identified using the Wilcoxon rank-sum test, and multiple testing correction was performed using the Benjamini–Hochberg method (adjusted *P* < 0.05). Subsequently, functional enrichment analyses of differentially expressed genes were performed using Gene Ontology (GO) and Kyoto Encyclopedia of Genes and Genomes (KEGG) databases. Both analyses were conducted using the clusterProfiler R package, with a significance threshold of adjusted *P* < 0.05 [29].

### Histology

Left kidneys were fixed in 4% paraformaldehyde for 24 h, paraffin-embedded, sectioned at 4 µm, and stained with hematoxylin–eosin (H&E), Masson’s trichrome, or periodic acid–Schiff (PAS). Slides were dehydrated, cleared, mounted, and examined for renal histopathology.

### Western blotting

Frozen kidney tissues were lysed in radioimmunoprecipitation assay buffer (RIPA) buffer with protease inhibitors. Protein concentrations were determined by bicinchoninic acid assay(BCA) (Servicebio, G2026). Equal protein amounts were separated by sodium dodecyl sulfate–polyacrylamide gel electrophoresis and transferred to polyvinylidene difluoride membranes, blocked, and incubated overnight at 4 °C with primary antibodies against CX3CL1 (Abclonal, A14198) and CCL2 (ABclonal, A23288). After washing, membranes were incubated with a horseradish peroxidase-conjugated secondary antibody, developed with enhanced chemiluminescence, and quantified using ImageJ.

### Quantitative real-time PCR

Total RNA was extracted from kidney tissues using a commercial kit, reverse-transcribed to cDNA, and subjected to quantative real-time PCR (qRT-PCR) using SYBR Green chemistry. β-Actin served as the reference gene. Relative expression levels were calculated by the 2^−ΔΔCt^ method. Primer sequences are provided in supplementary Table 2, see online supplementary material.

### Statistical analysis

Analyses followed the intention-to-treat principle. Continuous variables were expressed as mean ± SD and compared using independent or paired *t*-tests for normally distributed data, or Wilcoxon tests for non-normal data. Categorical variables were compared using χ² or Fisher’s exact tests. Potential confounders were adjusted by generalized linear models. Missing data were handled by multiple imputation (mice package in R).

A Bayesian linear regression model was further applied to evaluate the changes in 24hUTP, SCr, and eGFR at 24 weeks to validate the treatment effects. The analysis was performed using the stan_glm function in the R rstanarm package, with inference based on the dynamic Hamiltonian Monte Carlo sampler. Four chains were run with 20 000 iterations each (including 5 000 warm-up iterations). The effective samples size for key parameters reached 10 000, ensuring stability of posterior estimates. Weakly informative priors were specified as normal distributions with a mean of 0 and a SD of 5. Posterior distributions were summarized by the posterior mean, standard error, and 95% credible intervals, and Wilcoxon tests were used to further assess between-group differences. Model convergence was confirmed by the updated Rhat statistic (Rhat ≤ 1.01). Results were visualized using posterior density plots generated by ggplot2 [30].

For the animal study, analyses were conducted using SPSS 22.0. Data are presented as mean ± SD. One-way analysis of variance with least significant difference *post hoc* tests was used for normally distributed, homoscedastic data; otherwise, the Kruskal–Wallis test was applied. Significance was set at *P* < 0.05.

## Results

### Study population

**Figure 1.**
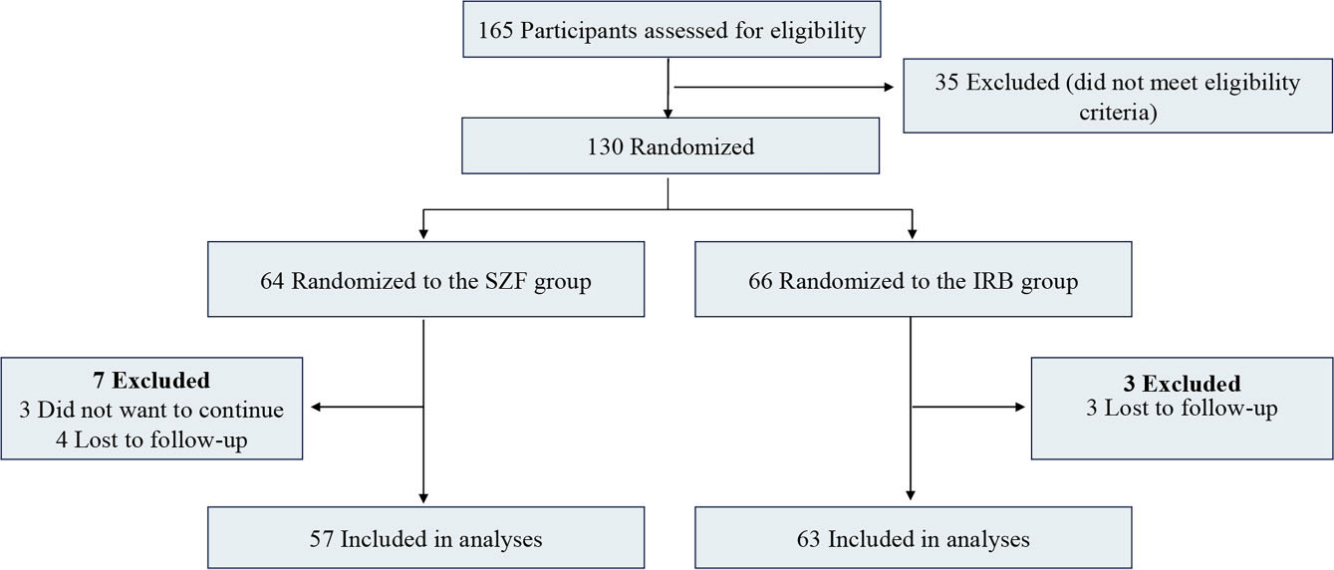
Enrollment, randomization, and follow-up of study participants.

The trial commenced recruitment of the first participant in July 2015. By December 2021, a total of 165 participants were screened across 11 medical centers in China. Of these, 130 patients were randomized, with 10 participants excluded after randomization (7 from the SZF group, and 3 from the IRB group). The remaining 120 patients comprised 57 in the SZF group and 63 in the IRB group (Fig. 1).

The average age of the remaining 120 participants in the SZF group was 59.75 ± 10.20 years, and 58.62 ± 11.05 years in the IRB group. Males accounted for 56.14% and 61.90% in the respective groups. Participants’ baseline characteristics were similar between groups (Table 1).

**Table 1.** Baseline characteristics of the participants in the full analysis set.

Characteristic^a^	SZF (*n* = 57)	IRB (*n* = 63)	*P* ^b^
Sex, No. (%)			
Female	25 (43.86)	24(38.10)	0.521
Male	32 (56.14)	39 (61.90)	
Age, mean (SD), years	60.02 (10.21)	58.79 (11.11)	0.786
BMI, mean (SD), kg/m^2^	25.04 (3.76)	25.82 (3.46)	0.105
Ethnicity, number (%)			
Han	57 (100.00)	61 (96.83)	0.497
Other	0 (0.00)	2 (3.17)	
Concomitant disease, number (%)			
Hypertension	36 (63.16)	38 (60.32)	0.749
Hyperlipidemia	15 (26.32)	11 (17.46)	0.240
Coronary atherosclerotic disease	7 (12.28)	11 (17.46)	0.427
Cerebrovascular disease	9 (15.79)	5 (7.94)	0.181
CKD stage, number (%)			
G1 stage	25 (43.86)	26 (41.27)	0.774
G2 stage	29 (50.88)	32 (50.79)	0.993
G3a stage	3 (5.26)	4 (6.35)	1.000
G3b stage	0 (0)	1 (1.59)	1.000
Concomitant medications, number (%)			
Metformin	10 (17.54)	14 (22.22)	0.522
α-Glucosidase Inhibitors	7 (12.28)	7 (11.11)	0.842
Sulfonylureas	7 (12.28)	4 (6.35)	0.261
Insulin	20 (35.09)	23 (36.51)	0.871
Calcium channel blockers	19 (33.33)	25 (39.68)	0.471
β-Blockers	3 (5.26)	2 (3.17)	0.667
Statins	13 (22.80)	12 (19.05)	0.613

^a^BMI, Body mass index (calculated as weight in kilograms divided by height in meters squared). G1 Stage: eGFR ≥ 90 mL/min/1.73 m². G2 Stage: eGFR 60–89 mL/min/1.73 m². G3a Stage: eGFR 45–59 mL/min/1.73 m². G3b Stage: eGFR 30–44 mL/min/1.73 m². ^b^*P*-Values were calculated using *t*-tests or Wilcoxon rank-sum tests for continuous variables, depending on the distribution of the data, and χ² tests or Fisher’s exact tests for categorical variables, depending on the expected frequencies.

### Primary outcome measure

For the primary outcome, the change in 24hUTP, revealed that after 24 weeks of treatment, the SZF group showed a change of −0.03 g/24 h [95% confidence interval (CI), −0.24 to 0.18], while the IRB group demonstrated a change of −0.08 g/24hUTP (95% CI, −0.30 to 0.14). No statistically significant difference was observed between the two groups (Table 2). Further analysis using a multivariate general linear model (GLM) revealed a lack of statistically significant differences in 24hUTP between the SZF and IRB groups (supplementary Table 3, see online supplementary material).

**Table 2.** Between-group analysis of outcome measures^a^.

	SZF (57)	IRB (63)	Difference (95%CI)^b^	*P*
**Primary outcomes**				
Change in 24hUTP				
Week 24	−0.03 (−0.24 to 0.18)	−0.08 (−0.30 to 0.14)	0.05 (−0.25 to 0.36)	0.61
**Secondary outcomes**				
**Change in SCr**				
Week 24	−5.15 (−9.73 to −0.56)	3.39 (−0.84 to 7.61)	−8.53 (−14.78 to −2.29)	<0.01
**Change in eGFR**				
Week 24	5.91 (1.80 to 10.01)	−1.67 (−5.18 to 1.84)	7.58 (2.16 to 13.00)	<0.01
**Change in FBG**				
Week 24	0.48 (−0.39 to 1.36)	0.00 (−0.82 to 0.81)	0.49 (−0.71 to 1.69)	0.53
**Change in HbA1c**				
Week 12	0.18 (−0.02 to 0.38)	0.33 (0.13 to 0.54)	−0.15 (−0.44 to 0.13)	0.20
Week 24	0.21 (−0.06 to 0.48)	0.24 (0.00 to 0.47)	−0.03 (−0.38 to 0.33)	0.63
**Change in TC**				
Week 12	0.26 (−0.06 to 0.58)	0.20 (−0.08 to 0.47)	0.06 (−0.36 to 0.48)	0.77
Week 24	−0.29 (−0.59 to 0.01)	0.27 (−0.05 to 0.59)	−0.56 (−1.00 to −0.12)	0.05
**Change in TG**				
Week 12	0.38 (−0.12 to 0.89)	0.37 (−0.28 to 1.02)	0.02 (−0.81 to 0.84)	0.76
Week 24	0.06 (−0.40 to 0.53)	−0.10 (−1.01 to 0.82)	0.16 (−0.86 to 1.19)	0.59
**Change in HDL-c**				
Week 12	−0.05 (−0.12 to 0.02)	−0.01 (−0.09 to 0.06)	−0.04 (−0.14 to 0.06)	0.34
Week 24	−0.09 (−0.17 to 0.00)	0.00 (−0.09 to 0.08)	−0.08 (−0.20 to 0.03)	0.13
**Change in LDL-c**				
Week 12	0.11 (−0.13 to 0.35)	0.16 (−0.04 to 0.36)	−0.05 (−0.36 to 0.26)	0.67
Week 24	−0.16 (−0.45 to 0.13)	0.25 (0.00 to 0.50)	−0.41 (−0.79 to −0.02)	0.07
**Change in SBP**				
Week 24	−0.19 (−2.80 to 2.41)	0.09 (−2.27 to 2.45)	−0.28 (−3.80 to 3.24)	0.60
**Change in DBP**				
Week 24	−3.89 (−6.21 to −1.57)	−1.61 (−3.75 to 0.53)	−2.28 (−5.44 to 0.89)	0.32
**Improvement of TCM syndromes**				
Overall clinical efficiency	51 (89.47%)	40 (63.49%)		<0.001
Clinical control	6 (10.53%)	2 (3.17%)		0.15
Significant effect	11 (19.30%)	9 (14.29%)		0.46
Effective	34 (59.65%)	29 (46.03%)		0.14
Ineffective	6 (10.53%)	23 (36.51%)		<0.001

^a^SBP, Systolic blood pressure; DBP, diastolic blood pressure. ^b^Continuous variables are presented as mean (95% CI) and were analyzed using independent samples *t*-tests or Wilcoxon rank-sum tests, depending on data distribution. Categorical variables are expressed as percentages (*n*) and were compared using χ² tests or Fisher’s exact tests, depending on the expected frequencies.

### Secondary outcome measures

The secondary outcomes revealed that after 24 weeks of treatment, the change in SCr was −5.15 (95% CI, −9.73 to −0.56) in the SZF group and 3.39 (95% CI, −0.84 to 7.61) in the IRB group, with a statistically significant difference between the two groups. Similarly, the change in eGFR was 5.91 (95% CI, 1.80 to 10.01) in the SZF group compared to −1.67 (95% CI, −5.18 to 1.84) in the IRB group, also showing a statistically significant difference (Table 2). Further analysis using a multivariate GLM revealed the improvement in SCr was significantly greater in the SZF group compared to the IRB group (9.09 [95% CI 2.93, 15.26]; *P* < 0.05; supplementary Table 3). Likewise, the improvement in eGFR was superior in the SZF group (−8.02 (95% CI, −13.18, −2.86); *P* < 0.01; supplementary  Table 3).

The change in TC was −0.29 (95% CI, −0.59 to 0.01) in the SZF group and 0.27 (95% CI, −0.05 to 0.59) in the IRB group, suggesting that TCM in the SZF group tended to decrease compared with the IRB group (*P* = 0.048). Additionally, after 24 weeks, the overall effective rate of TCM syndrome improvement was 89.47% in the SZF group and 63.49% in the IRB group, with a statistically significant difference between the groups (Table 2).

### Bayesian analysis

**Figure 2.**
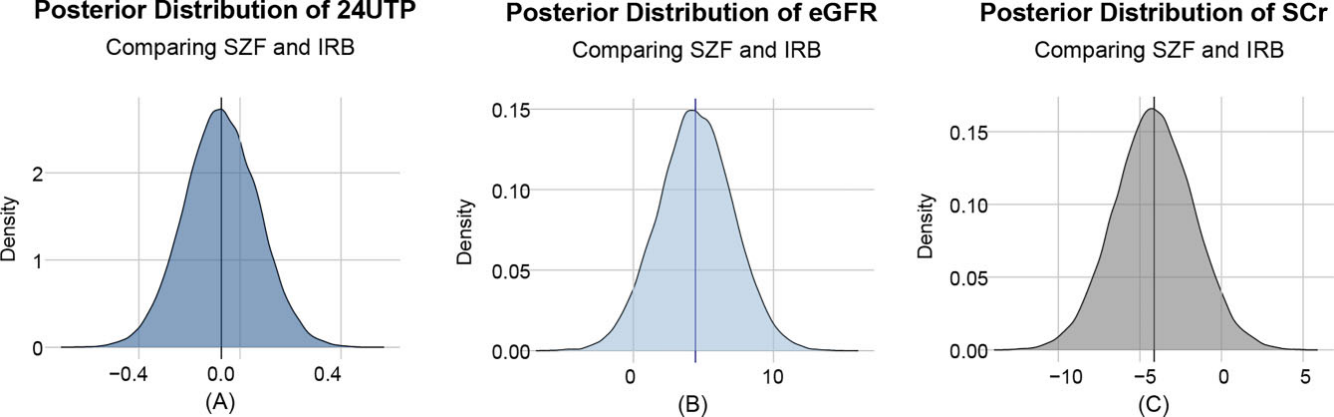
Bayesian analysis of the outcomes (using weakly informative priors). The absolute difference and full posterior probability distributions of changes in 24hUTP (**A**), eGFR (**B**), and SCr (**C**). The vertical lines indicate the mean values (used as the point estimate) and the areas highlighted indicate the percentile-based 95% credible intervals.

To further evaluate and validate the effects of SZF intervention, Bayesian analysis was performed to compare the changes in 24hUTP, SCr, and eGFR after 24 weeks of treatment. The results showed that the estimated absolute differences between the SZF and IRB groups were −0.05 (95% CI, −0.36 to 0.26) for 24hUTP, −5.81 (95% CI, −10.57 to −1.02) for SCr, and 6.06 (95% CI, 0.72 to 11.37) for eGFR. These findings indicate that while there was no significant difference in 24hUTP change between the two groups, significant improvements in SCr and eGFR were observed in the SZF group. The posterior distribution for the estimated 24hUTP change was symmetrically centered around zero, reflecting no clear advantage or disadvantage for either group in this outcome. In contrast, the probability that the SZF group reduced SCr by more than zero was >97.5%, suggesting a high likelihood of clinical benefit in lowering SCr. Similarly, the probability that the SZF group increased eGFR by more than zero was also >97.5%, indicating a strong potential for clinical benefit in improving eGFR (Fig. 2).

### Safety and tolerability

The safety data in this study showed that both treatments were generally well tolerated for patients with DKD. A total of nine AEs were reported by all participants, including five gastrointestinal events, one dermatologic event, and three edema events (supplementary Table 4, see online supplementary material). AEs were reported by 5 participants (8.77%) in the SZF group and 4 participants (6.35%) in the IRB group. Overall, there was no significant difference in the incidence of AEs between the two groups (*P* = 0.73).

### Regulation of Inflammatory proteins by SZF

**Figure 3.**
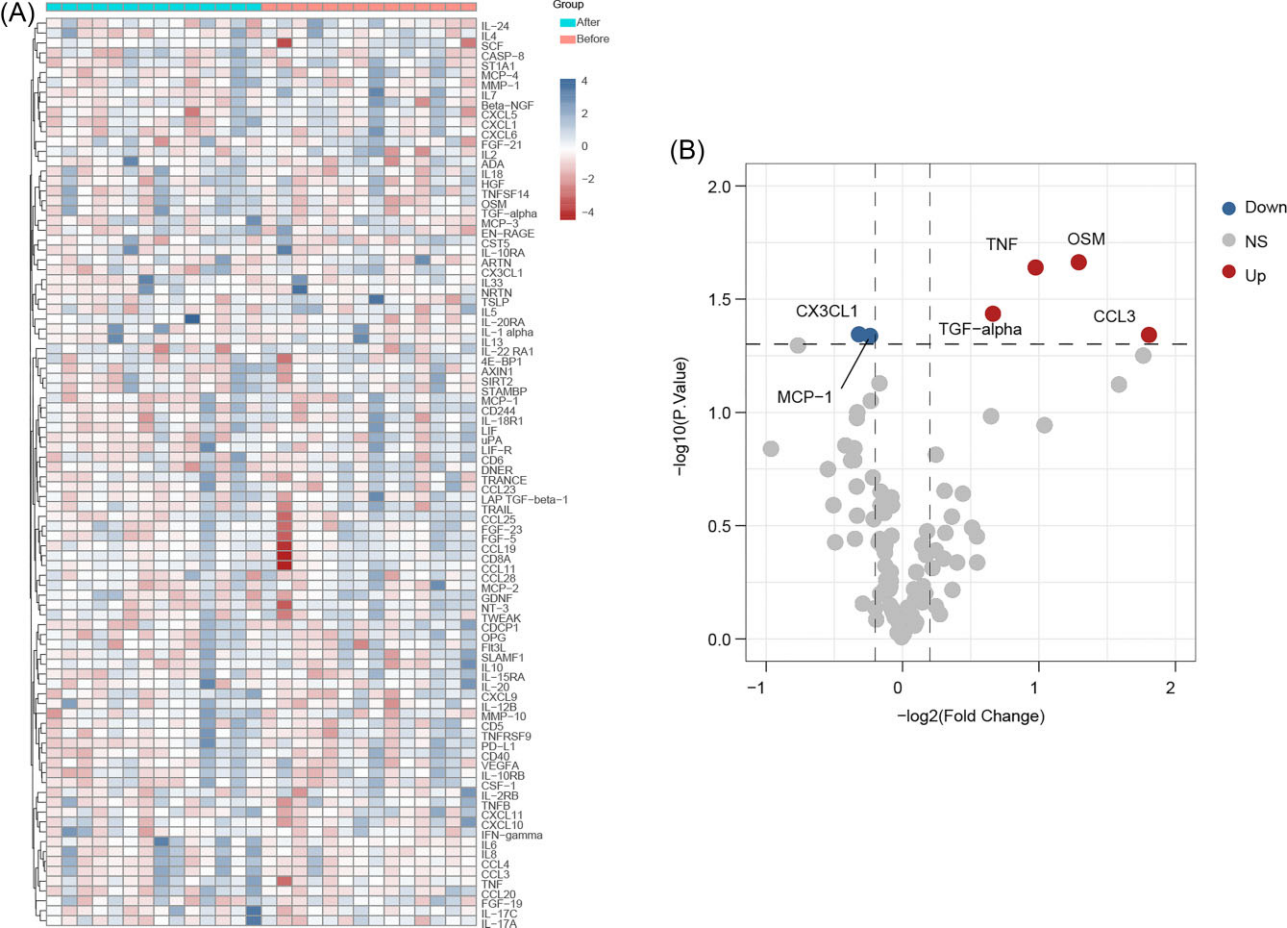
Regulation of inflammatory proteins by SZF. (**A**) Heatmap showing Olink proteomics profiling results. (**B**) Volcano plot of differentially expressed proteins.

Using Olink analysis, we compared the expression levels of 92 inflammation-related proteins before and after SZF treatment (Fig. 3A). Six differentially expressed proteins were identified, among which CX3CL1 and MCP-1 were significantly downregulated after treatment (Fig. 3B), suggesting they may be key targets mediating the anti-inflammatory effects of SZF.

### Effects of SZF on renal function in KK-Ay mice

**Figure 4.**
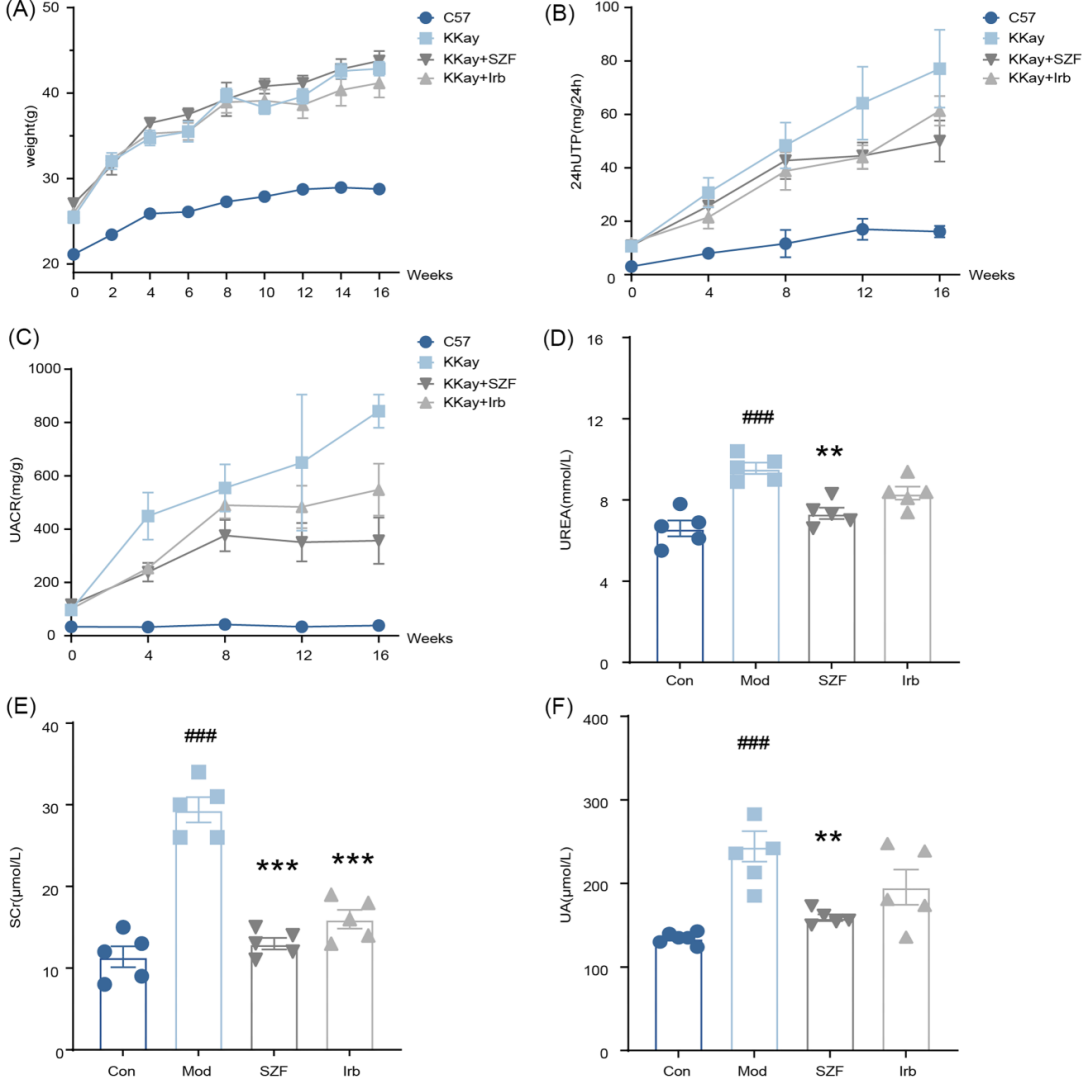
SZF intervention improves renal function parameters in DKD mice. (**A**) Body weight changes throughout the experiment. Changes in (**B**) 24hUTP, (**C**) urinary UACR, (**D**) serum urea levels, (**E**) SCr levels, and (**F**) serum UA levels over time. *n* = 5 per group. Compared with the Sham group: ###*P* < 0.001. Compared with the model group: **P < 0.01; ***P < 0.001.

To evaluate the protective effects of SZF in DKD mice, we monitored body weight and multiple renal function parameters. Compared with the normal control group, KK-Ay mice exhibited a significant increase in body weight. SZF treatment showed a similar weight-gain pattern to the model group, indicating that body weight was not markedly affected by the intervention (Fig. 4A). From week 4 onward, 24hUTP and urinary albumin-to-creatinine ratio (UACR) in the model group progressively increased, peaking at week 16 and remaining significantly higher than in the C57 group. SZF treatment markedly reduced both 24hUTP and UACR compared with the model group (Fig. 4B–C). For urea, SCr, and uric acid (UA), all three indicators were significantly elevated in the model group compared with controls (Fig. 4D–F). SZF intervention lowered urea and UA levels, with a particularly pronounced decrease in SCr. Collectively, these results indicate that SZF effectively attenuates DKD-associated renal dysfunction, improving proteinuria, UA levels, and other renal function markers.

### Single-cell transcriptomics reveals that SZF modulates CX3CL1 and MCP-1 expression

**Figure 5.**
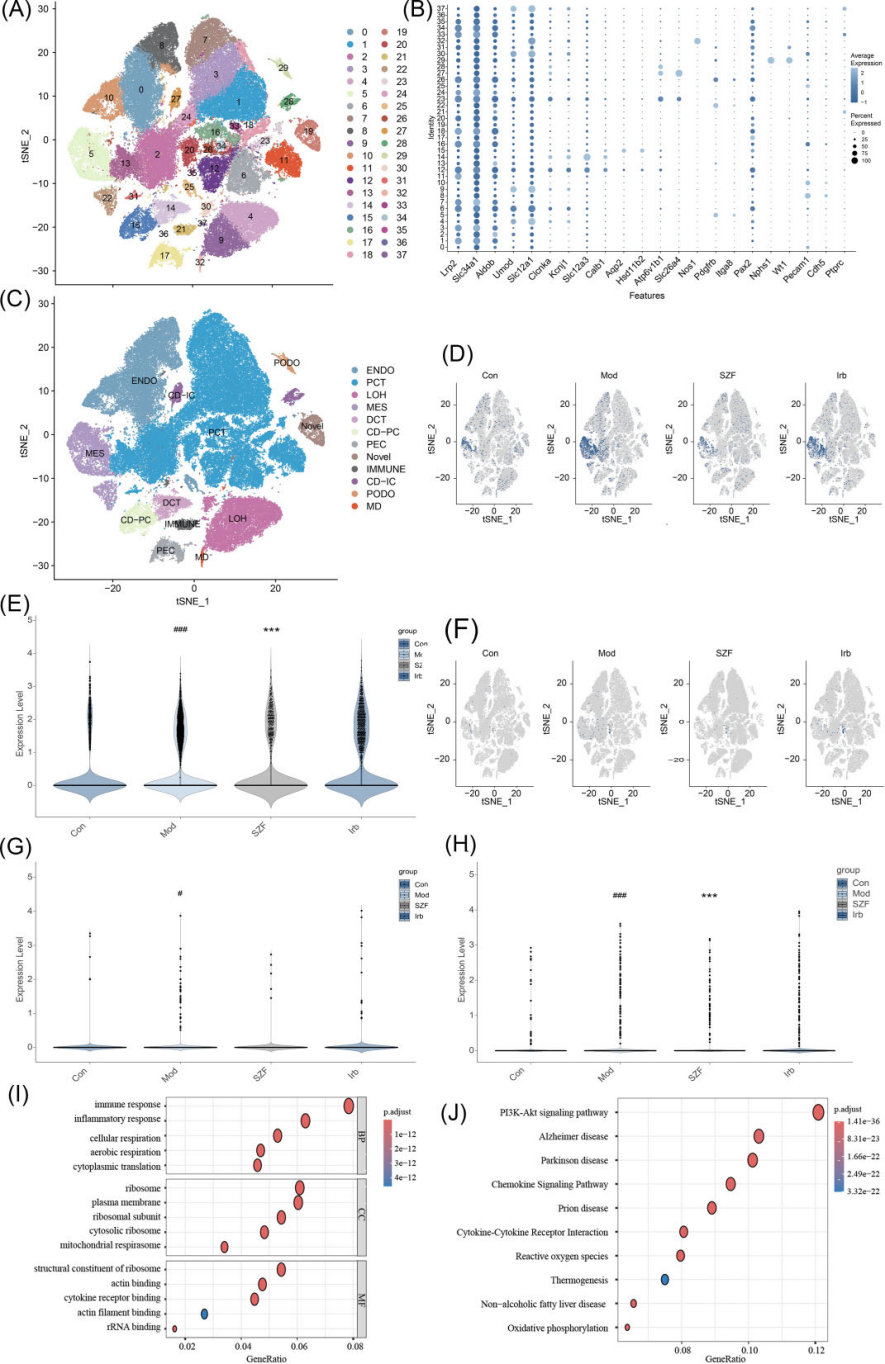
Single-cell transcriptomics reveals that SZF modulates CX3CL1 and MCP-1 expression. (**A**) t-SNE plot showing 38 identified cell clusters representing distinct kidney cell populations. (**B**) Dot plot displaying the expression patterns of selected canonical marker genes for cell-type annotation. (**C**) Annotation of 11 major kidney cell types and one unclassified cluster based on typical marker genes. (**D**) Feature plot showing CX3CL1 expression across different cell types. (**E**) Violin plot of CX3CL1 expression in MES. (**F**) Feature plot showing MCP-1 expression across different cell types. (**G**) Violin plot of MCP-1 expression in MES. (**H**) Violin plot of MCP-1 expression in PCT. (**I**) GO enrichment analysis results. (**J**) KEGG pathway enrichment analysis results.

Using scRNA seq, we characterized the cellular composition of mouse kidney tissue and the expression patterns of CX3CL1 and MCP-1. t-SNE analysis identified 38 distinct cell clusters (Fig. 5A). Based on canonical marker genes, 11 major cell types were annotated, including proximal tubule cells (PCT), loop of Henle cells (LOH), distal convoluted tubule cells (DCT), collecting duct principal cells (CD-PC), collecting duct intercalated cells (CD-IC), macula densa cells (MD), mesangial cells (MES), parietal epithelial cells (PEC), podocytes (PODO), endothelial cells (ENDO), and immune cells (Fig. 5B and C).

CX3CL1 expression was markedly elevated in endothelial cells of the model group, and SZF treatment significantly reduced its expression in this cell population (Fig. 5D and E). MCP-1 expression was increased in MES and PCT of the model group, with SZF treatment notably decreasing MCP-1 expression in PCT (Fig. 5F–H). GO and KEGG pathway enrichment analyses were added, showing that SZF regulates inflammatory response, chemokine signaling, and immune-related pathways, consistent with the downregulation of CX3CL1 and MCP-1(Fig. 5I and J).

### SZF alleviates renal pathological injury and suppresses CX3CL1 and MCP-1 expression

**Figure 6.**
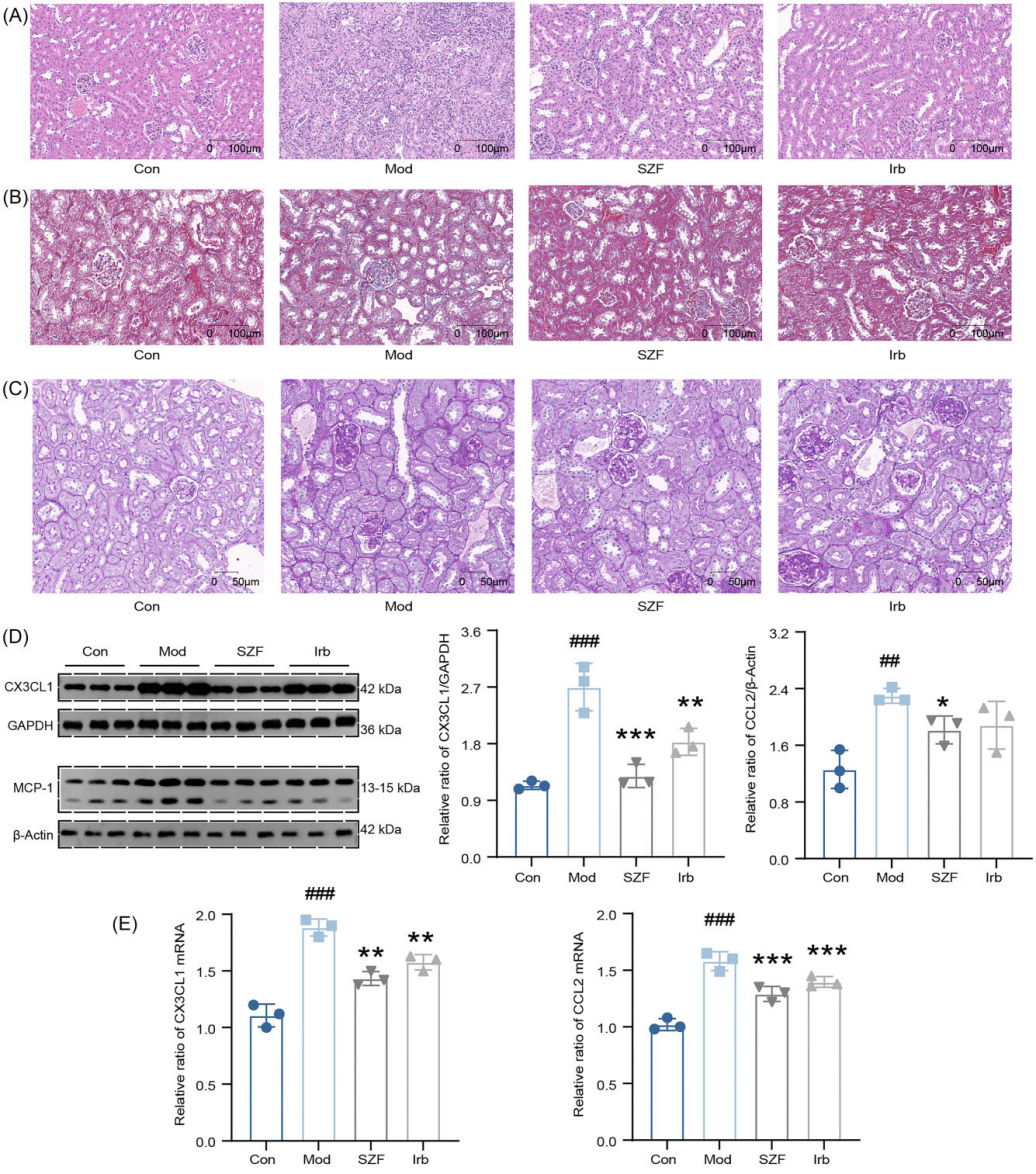
SZF ameliorates renal injury in mice by regulating CX3CL1 and MCP-1 expression. (**A**) Representative images of H&E staining; (**B**) Masson’s trichrome staining; (**C**) PAS staining; (**D**) western blot analysis of CX3CL1 and MCP-1; (**E**) RT-qPCR analysis of CX3CL1 and MCP-1. *n* = 3. Compared with the Sham group: ##*P* < 0.01; ###*P* < 0.001. Compared with the model group: *P < 0.05; **P < 0.01; ***P < 0.001.

Compared with the control group, kidneys from model mice exhibited severe structural damage, characterized by disorganized glomerular architecture and vacuolar degeneration of tubular epithelial cells. SZF treatment markedly attenuated these pathological alterations (Fig. 6A). Masson’s trichrome staining revealed pronounced interstitial collagen fiber deposition in the model group, indicating significant renal interstitial fibrosis (Fig. 6B). In the SZF-treated group, collagen accumulation was notably reduced, with a smaller fibrotic area. PAS staining further demonstrated glomerular basement membrane thickening and mesangial expansion in the model group, both of which were substantially improved by SZF (Fig. 6C).

qRT-PCR and western blot analyses showed that SZF significantly decreased both mRNA and protein expression levels of CX3CL1 and MCP-1, suggesting that its renoprotective effects may be mediated, at least in part, through suppression of CX3CL1- and MCP-1-driven inflammatory pathways (Fig. 6D and E).

## Discussion

The present multicenter, randomized, double-blind, active comparator, parallel-controlled trial demonstrated that after 24 weeks of intervention, the therapeutic efficacy of SZF in reducing the 24hUTP difference (endpoint-baseline) in DKD patients with macroalbuminuria was not significantly different from the active control drug, IRB. However, SZF significantly reduced SCr levels, improved eGFR levels, and alleviated TCM syndromes in DKD patients with macroalbuminuria, outperforming IRB in these areas. Bayesian analysis further indicated that SZF is more likely to provide clinical benefits in lowering SCr and increasing eGFR. Additionally, SZF appeared to have a favorable effect on lipid profiles compared to IRB. SZF also exhibited a good safety profile in these patients. In addition to the clinical outcomes, the multi-omics analyses provided further mechanistic evidence supporting the renoprotective effects of SZF. Integrated Olink proteomic and scRNA-seq analyses revealed that SZF significantly downregulated the proinflammatory mediators CX3CL1 and MCP-1, which were primarily expressed in renal endothelial cells and mesangial and proximal tubular cells, respectively. Consistent with these findings, GO and KEGG enrichment analyses demonstrated that SZF mainly modulates the chemokine signaling pathway, cytokine–cytokine receptor interaction, and the PI3K–Akt signaling pathway, all of which are closely associated with inflammatory and fibrotic processes in DKD. These mechanistic insights align with the clinical observations of reduced SCr and increased eGFR, suggesting that the improvement in renal function following SZF treatment may result from the direct modulation of inflammatory and stress-response pathways within renal parenchymal cells.

Clinical and basic research have extensively studied the components of SZF to understand their effects on DKD. Huangqi (*Astragalus mongholicus Bunge*), a widely used herb for treating DKD, has been shown in systematic review and meta-analysis to improve creatinine clearance and reduce albuminuria in patients [31]. A study using *in vitro* and *in vivo* experiments demonstrated that its main active component, astragaloside IV, alleviates mitochondrial dysfunction by activating the nuclear factor erythroid 2–related factor 2 (Nrf2)-antioxidant response element/mitochondrial transcription factor A signaling pathway, thereby counteracting oxidative stress-induced diabetic kidney injury and podocyte apoptosis [32]. Dahuang (*Rheum palmatum L*.), another key ingredient, has demonstrated efficacy in reducing albuminuria and improving renal function with minimal adverse effects [33]. The active compound rhein exhibits potent nephroprotective properties by regulating the Rac1/NOX1/beta-catenin axis to suppress ferroptosis and epithelial–mesenchymal transition, thus alleviating DKD [34]. Danshen (*Salvia miltiorrhiza Bunge*) contains major active compounds, including lipophilic tanshinones such as tanshinone I and tanshinone II, and water-soluble phenolic acids like salvianolic acid A and salvianolic acid B. Meta-analysis indicates that tanshinone IIA, as an adjuvant therapy, enhances the therapeutic efficacy in DKD [35]. Another study further revealed that salvianolic acid B and tanshinone IIA synergistically improve early-stage DKD by regulating the PI3K/Akt/NF-κB signaling pathway [36]. Additionally, research suggests that the combination of Danshen (*Salvia miltiorrhiza Bunge*) and Huangqi (*Astragalus mongholicus Bunge*) ameliorates DKD through the brain–kidney axis by increasing the abundance of *Akkermansia muciniphila* and *Lactobacillus murinus*, and modulating pathways related to sphingolipid and glycerophospholipid metabolism, which are associated with glucose and lipid metabolism [37]. A meta-analysis has shown that Shuizhi (*Whitmania pigra Whitman*) combined with Western medicine is more effective in delaying DKD progression and protecting renal function compared to Western medicine alone [38]. A mechanistic study indicates that freeze-dried Shuizhi (*Whitmania pigra Whitman*) exerts renoprotective effects by inhibiting oxidative stress and the production of inflammatory cytokines, as well as suppressing the activation of the JAK2/STAT1/STAT3 signaling pathway in renal tissues of DKD rats [39]. Yinyanghuo (*Epimedium brevicornu Maxim*.) contains >260 identified chemical compounds, including flavonoids, polysaccharides, and phytosterols, with icariin being the most abundant. A study has shown that icariin inhibits oxidative stress through G protein–goupled estrogen receptor-mediated, p62-dependent kelch-like ECH-associated protein 1 degradation and Nrf2 activation, preventing extracellular matrix accumulation and improving experimental DKD [40]. Leonurine, a unique alkaloid found in Yimucao (*Leonurus japonicus Houtt.)*, has been shown to ameliorate renal fibrosis in unilateral ureteral obstruction mice by suppressing the TGF-β and NF-κB signaling pathways [41].

ACEI/ARB are the first-line treatments for patients with DM complicated by CKD and hypertension, especially those presenting with albuminuria. In this study, IRB was selected as the active control drug, with findings indicating that SZF’s efficacy in reducing albuminuria was comparable to that of IRB. Despite pharmacological interventions, DKD often progresses inexorably to ESRD. A prospective study involving 10 184 community-dwelling individuals aged ≥66 years assessed kidney function decline. Results revealed that eGFR decline was most pronounced in patients with diabetes, with annual declines of 2.1 ml/min/1.73 m² (95% CI, 1.8–2.5) in women and 2.7 ml/min/1.73 m² (95% CI, 2.3–3.1) in men [42]. Currently recommended first-line therapies, including ACEI/ARB and sodium–glucose co-transporter-2 (SGLT2) inhibitors, can delay eGFR decline but have not demonstrated improvements in eGFR. For instance, losartan reduces the annual rate of eGFR decline by 18% [7], while empagliflozin decreases the average eGFR decline from 1.67 ml/min/1.73 m² in the placebo group to 0.19 ml/min/1.73 m² in the treatment group when added to ACEI/ARB therapy [43]. These findings highlight the limited capacity of mainstream therapies to improve glomerular filtration rates in DKD patients. Despite the 24-week intervention duration, this study demonstrates that SZF has the potential to improve eGFR and may also enhance lipid profiles, making it a promising candidate for further investigation.

The TCM syndrome score is a method based on TCM theory that evaluates and analyzes patients' symptoms and signs through scoring. On the one hand, TCM syndrome scores can reflect changes in patients' syndromes; on the other hand, they can provide insight into patients' quality of life [44]. For patients diagnosed with chronic non-communicable diseases, improving quality of life is closely linked to their overall health status [45]. This study demonstrated that SZF significantly improves TCM syndrome scores in DKD patients with macroalbuminuria, playing a positive role in enhancing their quality of life. Moreover, our study found no significant difference in the proportion of AEs between the SZF and IRB groups. Therefore, SZF appears to be a safe treatment option for DKD patients, though further evaluation is needed.

Olink proteomic profiling revealed that SZF treatment markedly downregulated CX3CL1 and MCP-1, both of which are closely associated with renal inflammation and fibrotic progression in diabetes [46, 47]. scRNA-seq further localized these inflammatory changes to specific renal cell types: CX3CL1 was abundantly expressed in endothelial cells of KK-Ay mice, suggesting a role in promoting monocyte adhesion and transglomerular migration. SZF administration significantly reduced endothelial CX3CL1 expression, indicating a potential mechanism of restoring endothelial barrier integrity [48]. MCP-1 expression was predominantly elevated in mesangial cells and proximal tubular cells, reflecting its role in mesangial proliferation, pro-inflammatory cytokine release, and tubulointerstitial inflammation [49, 50]. SZF intervention markedly suppressed MCP-1 overexpression in proximal tubular cells, suggesting that it may alleviate the tubulointerstitial inflammatory microenvironment, reduce immune cell infiltration, and thereby attenuate renal inflammation and fibrosis. Functionally, these molecular changes were accompanied by significant improvements in proteinuria, SCr, and histopathological features. Masson’s trichrome and PAS staining confirmed that SZF reduced interstitial collagen deposition, mesangial expansion, and glomerular basement membrane thickening, consistent with its anti-inflammatory and anti-fibrotic potential, via targeting CX3CL1 and MCP-1. Notably, SZF improved renal function without affecting body weight, suggesting that its benefits are likely mediated through direct modulation of inflammatory and fibrotic pathways rather than metabolic alterations.

From a translational medicine perspective, the signaling pathways regulated by SZF are highly consistent with the mechanisms targeted by current renoprotective strategies. Inflammation and fibrosis are widely recognized as key pathological processes driving the onset, progression, and transition of DKD to ESRD. The development of renal inflammation involves the release of multiple proinflammatory mediators, sustained activation of inflammatory signaling cascades, and recruitment and infiltration of immune cells, all of which collectively contribute to glomerular and tubulointerstitial injury and fibrosis [51, 52]. Our findings suggest that SZF may exert additional functional benefits by directly modulating inflammatory and stress-response signaling within renal parenchymal cells, rather than relying solely on hemodynamic or glycemic regulation. This mechanistic convergence with existing therapeutic paradigms, together with the favorable safety profile observed in clinical trials, indicates that the anti-inflammatory properties of SZF have meaningful translational potential for the management of DKD.

This study has several limitations. First, the clinical intervention period was only 24 weeks, and the study did not adopt “hard endpoints” such as ESRD, dialysis, or renal replacement therapy as the primary outcomes; therefore, it was insufficient to fully evaluate the long-term benefits of SZF. Future studies should consider continuous follow-up of all enrolled patients to assess long-term efficacy. Second, 24hUTP was used as a comprehensive indicator for proteinuria assessment. Although it provides an overall evaluation of protein excretion, 24-hour urine collection requires high patient compliance and may be affected by incomplete collection, potentially reducing the reliability of the results. Because UACR testing was not uniformly implemented at all participating centers during the early phase of the study, it could not be included in the overall analysis. In future studies, UACR will be adopted as the primary endpoint to improve comparability, enhance clinical applicability, and align with current nephrology research standards. In addition, since patients with SCr ≤ 133 μmol/L were enrolled, the study population mainly represented the early stages of DKD. This inclusion criterion was selected primarily for safety considerations and to ensure the evaluability of therapeutic efficacy while minimizing confounding from patients with severe renal impairment. Nevertheless, the generalizability of these findings to more advanced DKD populations requires further validation in future studies. Third, although the mechanistic investigation identified CX3CL1 and MCP-1 as key inflammatory mediators regulated by SZF, other potential targets and signaling pathways have not yet been systematically validated. Furthermore, functional rescue experiments—such as gene knockdown, overexpression, or pharmacological inhibition—are still lacking to directly confirm the causal relationship between SZF-mediated modulation of CX3CL1/MCP-1 and its renoprotective effects. Fourth, only male KK-Ay mice were used in the animal experiments, and potential sex-related differences were not evaluated, which may limit the generalizability of the findings. Finally, although multi-omics analyses provided valuable insights into the molecular mechanisms of SZF, the translational relevance of these findings requires further validation in patient-derived samples or humanized models.

Future work will focus on expanding experimental verification through genetic and pharmacological approaches, incorporating both sexes in animal studies, and integrating molecular biomarkers into long-term clinical trials to strengthen the causal and translational evidence for SZF in DKD. In addition, future studies will consider extending the follow-up period and conducting larger, multicenter clinical trials to further verify the long-term efficacy and safety of SZF, providing a more comprehensive evaluation of its clinical applicability and therapeutic potential.

## Conclusion

In conclusion, SZF appears to be comparable to IRB in reducing 24hUTP levels in DKD patients with macroalbuminuria, while demonstrating superior efficacy in improving eGFR. SZF has shown good safety and efficacy, making it a promising adjunctive treatment for DKD patients with macroalbuminuria.

## Supplementary Material

pbaf031_Supplemental_File

## Notes

Trial registration: Chinese Clinical Trial Registry ChiCTR-ICR-15006311
