# Supplementary material for: Randomized controlled clinical trial of Shenzhuo Formula in the treatment of macroalbuminuria in diabetic kidney disease and its inflammation-modulating mechanisms
Source: Precis Clin Med. 2025 Nov 14;8(4):pbaf031. doi: 10.1093/pcmedi/pbaf031 (PMC12699223; doi:10.1093/pcmedi/pbaf031)
Supplement: pbaf031_Supplemental_File [file pbaf031_supplemental_file.docx]

**Supplementary Data**

**Supplementary Table 1. Composition and proportion of SZF.**

| **Chinese name** | **Latin name** | **Weight, g** | **Part used** |
| --- | --- | --- | --- |
| Da Huang | Rheum palmatum L. | 6 | Root and rhizome |
| Shui Zhi | Whitmania pigra Whitman | 3 | Dried whole body |
| Huang Qi | Astragalus mongholicus Bunge | 30 | Root |
| Dan Shen | Salvia miltiorrhiza Bunge | 15 | Root and rhizome |
| Yin Yang Huo | Epimedium brevicornu Maxim. | 6 | Aerial parts |
| Yi Mu Cao | Leonurus japonicus Houtt. | 15 | Aerial parts |

Note: The plant names have been verified through http://www.worldfloraonline.org, with the access date mentioned. The animal names have been confirmed through https://ydz.chp.org.cn, with the access date of February 16, 2025.

**Supplementary Table 2. Primer sequences of each gene.**

| **Gene** | **Primer sequence （5′→3′）** | **Temp（℃）** |
| --- | --- | --- |
| CX3CL1 | Forward：AACTTCCGAGGCACAGGATG  Reverse：CCAAACGGTGGTGGAGATGT | Forward：60.04  Reverse：60.25 |
| CCL2 | Forward：TGACCCCAAGAAGGAATGGG  Reverse：ACCTTAGGGCAGATGCAGTT | Forward：59.30  Reverse：59.00 |
| β-Actin | Forward：TCAGCAAGCAGGAGTACGATG  Reverse：AAAACGCAGCTCAGTAACAGT | Forward：60.13  Reverse：58.44 |

**Supplementary Table 3. Generalized Linear Model Analysis of Factors Influencing Changes in 24hUTP, SCr, and eGFR**

| **Factors** | **Coefficient (95% Confidence Interval)** | ***P* Value** |
| --- | --- | --- |
| **Change of 24hUTP** |  |  |
| Age, yrs | -0.01 (-0.02, 0.01) | 0.430 |
| Sex (female/male) | -0.27 (-0.58, 0.05) | 0.109 |
| Baseline | -0.11 (-0.32, 0.10) | 0.348 |
| Group | -0.05 (-0.35, 0.26) | 0.769 |
| Metformin | -0.13 (-0.52, 0.27) | 0.550 |
| α-Glucosidase Inhibitors | 0.15 (-0.35, 0.65) | 0.577 |
| Sulfonylureas | -0.04 (-0.61, 0.52) | 0.769 |
| Insulin | -0.10 (-0.43, 0.23) | 0.557 |
| Calcium channel blockers | -0.24 (-0.57, 0.08) | 0.191 |
| β-blockers | 0.25 (-0.54, 1.05) | 0.546 |
| Statins | 0.04 (-0.35, 0.42) | 0.710 |
| **Change of SCr** |  |  |
| Age, yrs | -0.03 (-0.33, 0.27) | 0.570 |
| Sex (female/male) | -3.29 (-10.23, 3.64) | 0.415 |
| Baseline | -0.14 (-0.31, 0.03) | 0.128 |
| Group | 9.09 (2.93, 15.26) | 0.013 |
| Metformin | -0.60 (-8.42, 7.21) | 0.682 |
| α-Glucosidase Inhibitors | -6.81 (-16.51, 2.89) | 0.191 |
| Sulfonylureas | 1.15 (-9.86, 12.16) | 0.712 |
| Insulin | 2.36 (-4.16, 8.88) | 0.527 |
| Calcium channel blockers | 1.14 (-5.48, 7.75) | 0.516 |
| β-blockers | 13.75 (-2.14, 29.65) | 0.129 |
| Statins | -3.454 (-11.08, 4.17) | 0.390 |
| **Change of eGFR** |  |  |
| Age, yrs | -0.29 (-0.57, -0.01) | 0.136 |
| Sex (female/male) | 3.69 (-1.64, 9.03) | 0.233 |
| Baseline | -0.27 (-0.43, -0.12) | 0.002 |
| Group | -8.02 (-13.18, -2.86) | 0.008 |
| Metformin | 0.05 (-6.46, 6.57) | 0.782 |
| α-Glucosidase Inhibitors | 4.66 (-3.47, 12.79) | 0.284 |
| Sulfonylureas | -3.62 (-12.78, 5.55) | 0.474 |
| Insulin | -2.20 (-7.62, 3.22) | 0.470 |
| Calcium channel blockers | -0.06 (-5.58, 5.45) | 0.650 |
| β-blockers | -3.45 (-11.07, 4.17) | 0.390 |
| Statins | 2.80 (-3.55, 9.15) | 0.395 |

Note: Abbreviations: 24hUTP ,24-hour urinary total protein; eGFR, estimated glomerular filtration rate, SCr, serum creatinine.

**Supplementary Table4. Adverse events in SZF and IRB groups.**

|  | **SZF (57)** | | **IRB (63)** | |
| --- | --- | --- | --- | --- |
|  | Participants | Events | Participants | Events |
| Any adverse events | 5(8.77%) | 5 | 4(6.35%) | 4 |
| Types of Adverse Events | | | | |
| Gastrointestinal events | 4(7.01%) | 4 | 1(1.59%) | 1 |
| Dermatologic events | 1(1.75%) | 1 | 0 | 0 |
| Edema events | 0 | 0 | 3(4.76%) | 3 |

Note: Categorical variables were analyzed using Fisher’s exact test.


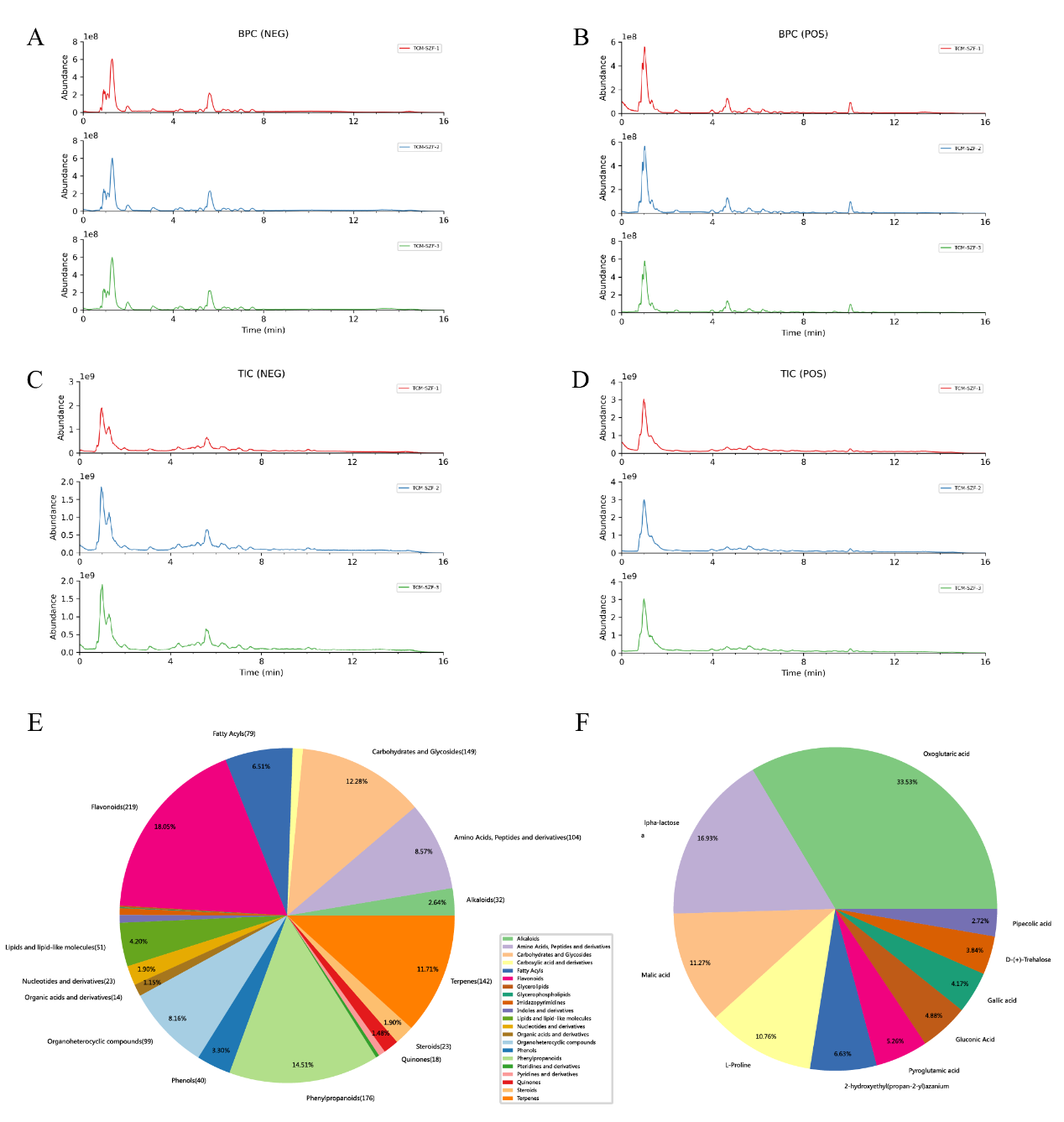


**Supplementary** **Figure 1.** The main chemical components of SZF were analyzed by liquid chromatography-mass spectrometry (LC-MS). (**A**) BPC chromatogram of three batches of SZF in negative ion mode. (**B**) BPC chromatogram of three batches of SZF in positive ion mode. (**C**) TIC chromatogram of three batches of SZF in negative ion mode. (**D**) TIC chromatogram of three batches of SZF in positive ion mode. (**E**) Distribution of component categories in SZF. (**F**) Top 10 components by content in SZF.
